# Traumatic Optic Neuropathy and Central Retinal Artery Occlusion Following Blunt Ocular Trauma

**DOI:** 10.4021/jocmr497w

**Published:** 2011-02-12

**Authors:** Tongabay Cumurcu, Selim Doganay, Soner Demirel, Cem Cankaya

**Affiliations:** a Inonu University School of Medicine, Department of Ophthalmology, Malatya, Turkey

## Abstract

We present a case as a rare sign of traumatic optic neuropathy and central retinal artery occlusion following blunt ocular trauma. A 10-year-old child suffered complete loss of the vision of one eye following a blunt ocular injury. He sustained an occlusion of the central retinal artery and traumatic optic neurupathy of the affected eye. Isolated cases of central retinal vessel occlusions and traumatic optic neurapathy following ocular blunt trauma are rare conditions. Clinicians to be aware of the potential for blunt ocular trauma to cause optic nerve damage and retinal vessel occlusions.

**Keywords:**

Blunt ocular trauma; Retinal artery occlusion; Traumatic optic neuropathy

## Introduction

Traumatic optic neuropathy following blunt or penetrating injury occurs with an incidence of 2% - 5% in facial trauma [1]. The mechanism of the injury could be direct mechanical compression of the optic nerve, this compression in combination with compression of the central retinal artery and traction, or the compression effect on the small nutrient vessels feeding the optic nerve. Compression forces transmitted to the orbital apex cause a compartment syndrome whereby compression leads to a vicious cycle of swelling and ischemia, release of free oxygen radicals, and damage to axons [2].

Previously, isolated cases of central retinal vessel occlusions and optic nerve damage following ocular trauma have been reported although as rare events [3-5].

We present a case as a rare sign of traumatic optic neuropathy and central retinal artery occlusion following blunt ocular trauma.

## Case Report

**Figure 1.**
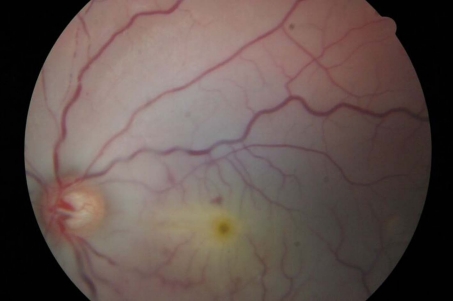
Pale macular area with a cherry-red spot in the left eye.

A 10-year-old child suffered a blunt ocular trauma to the left eye and noted total visual loss in the eye. Injury occurred while playing football. We saw him 12 hours post-trauma with visual acuity (VA) of no light perception (NLP) in the left eye. Anterior segment exam was unremarkable. Funduscopic examination was normal OS on the first day. A pale macular area with a cherry-red spot was noted in the left eye on the second day (Fig. 1) along with areas of retina whitening. The left optic disc was mildly pale and RAPD was positive. No perforation of the sclera or cornea was found. His right VA was found to be 20/20, and anterior segment and fundoscopic examination was normal OD during the following days. The intraocular pressure of each eye was 14 mmHg.

**Figure 2.**
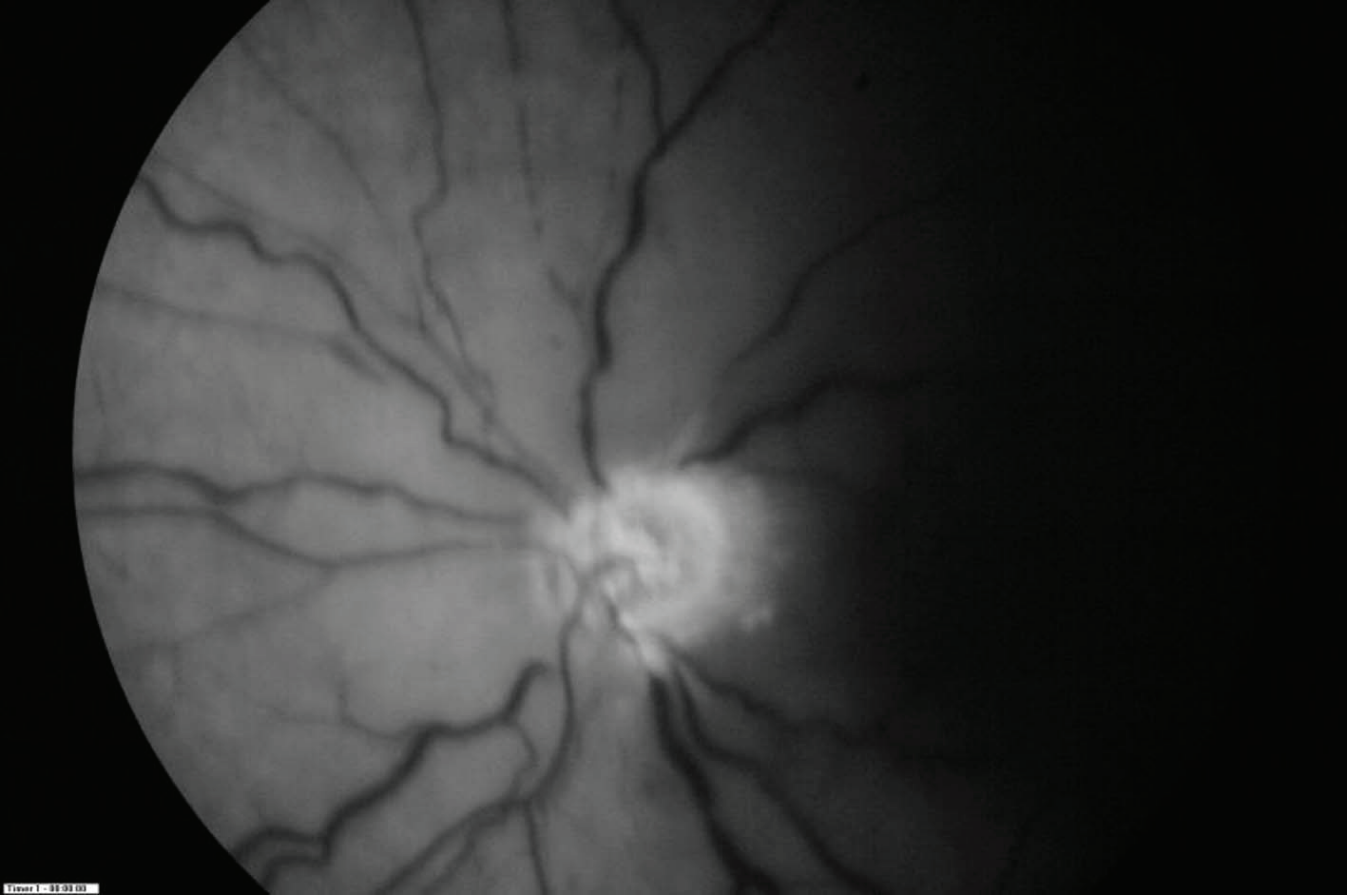
Fundus fluorescein angiogram in the left eye showed central retinal artery occlusion with capillary non-perfusion in early stage.

**Figure 3.**
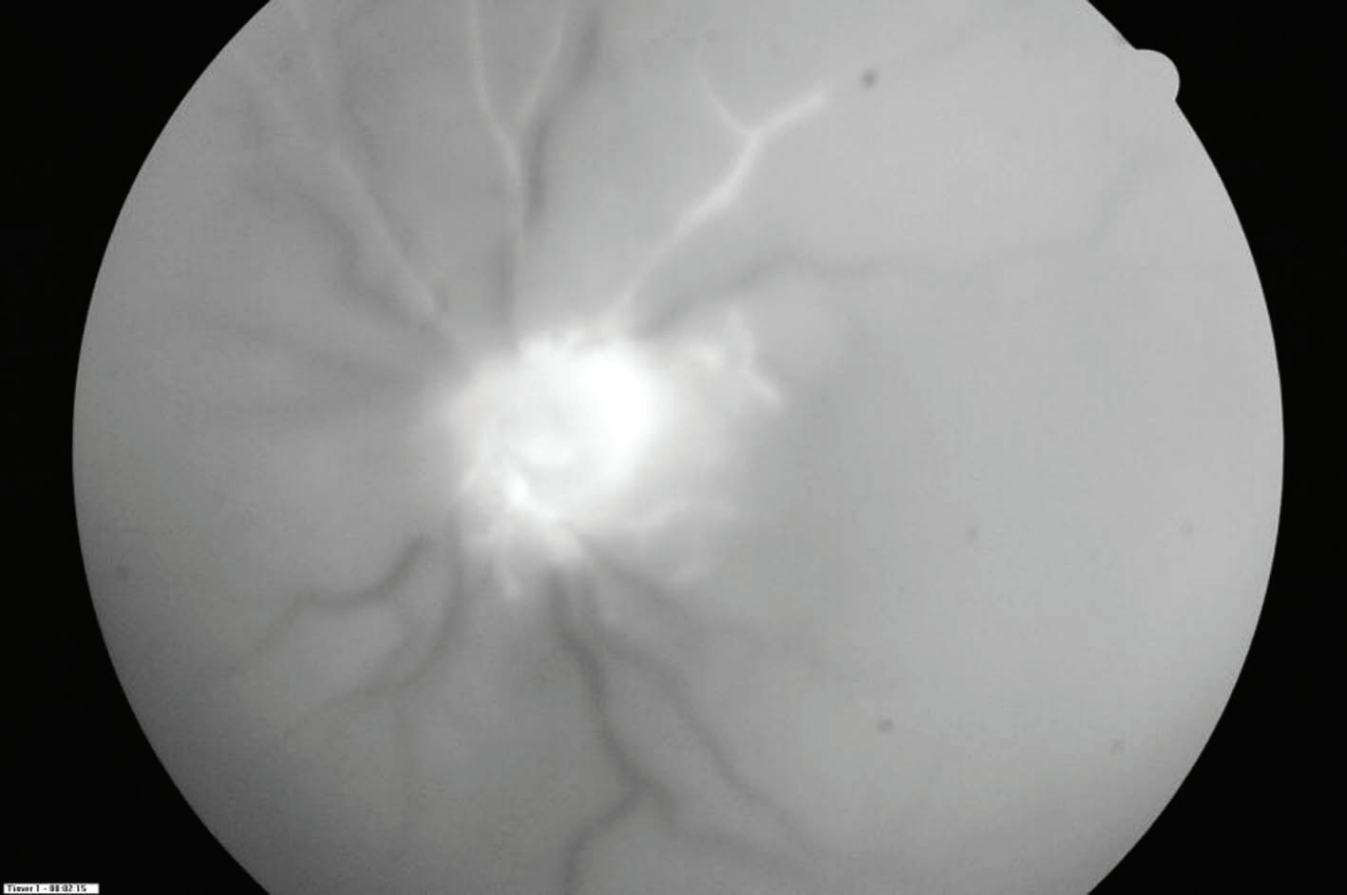
Late stage of fundus fluorescein angiogram showed hyperfluorescence of the optic disc in the left eye, also, occlusion of central retinal artery with markedly retarded influx of fluorescein.

Our patient had neither a familial history nor any clinical and laboratorial evidence of SLE or hemoglobinopathy. CT scan and MRI showed the edema of the optic nerve OS. The patient received intravenous bolus therapy with methylprednisolone for 72 h followed by oral prednisone. Fluorescein angiography of the left eye 1 week later demonstrated an area of retinal arterial occlusions with non-perfusion of the macula and retina. Choroidal perfusion was maintained (Fig. 2, 3). At 1-month follow-up VA was still NLP in the left eye.

## Discussion

Epidemiological studies have shown higher rates of ocular injury in male adults in the younger age group which may be due to trauma at work, secondary to attack, sports or road traffic accidents. It has been estimated that up to half a million people in the world are blind as a result of ocular injuries [6].

Also, our patient was a male in the younger age group and the blunt ocular trauma occured while playing football.

The pathophysiology for this occlusion may involve the disruption of the endothelium from acute stretching of the retinal vessels due to the sudden deformation of the eye. Intimal disruption is a well-documented cause of vessel occlusion by thrombosis in other areas of the body. Because the intima is the least elastic layer of the vessel wall, direct injury or even stretching of the vessel tends to tear and disrupt this innermost layer, exposing the subintimal tissue to the bloodstream. Platelets aggregate around the damaged endothelium, initiating the coagulation cascade and resulting in thrombus formation. Arterial thrombosis and occlusion may be facilitated by local vasospasm of the injured vessel, a natural homeostatic response to trauma [4, 7, 8].

Usually, visual loss ocurred over hours, and some cases vision continued to deteriorate for several weeks. Our case suffered complete loss of vision in the left eye from the time of his blunt ocular trauma. This may have been the result of an associated optic nerve injury [3, 4].

Traumatic retinal arterial occlusion in patient with hemoglobinopathy and systemic lupus erithematosus (SLE) has been reported, but our case had neither a familial history nor any clinical and laboratorial evidence of SLE or hemoglobinopathy [9, 10].

This case highlights the need for clinicians to be aware of the potential for blunt ocular trauma to cause optic nerve damage and retinal vessel occlusions.

## References

[1] Holt GR, Holt JE (1983). Incidence of eye injuries in facial fractures: an analysis of 727 cases. Otolaryngol Head Neck Surg.

[2] Steinsapir KD, Goldberg RA (1994). Traumatic optic neuropathy. Surv Ophthalmol.

[3] Noble MJ, Alvarez EV (1987). Combined occlusion of the central retinal artery and central retinal vein following blunt ocular trauma: a case report. Br J Ophthalmol.

[4] Dalma-Weiszhausz J, Meza-de Regil A, Martinez-Jardon S, Oliver-Fernandez K (2005). Retinal vascular occlusion following ocular contusion. Graefes Arch Clin Exp Ophthalmol.

[5] Chong CC, Chang AA (2006). Traumatic optic nerve avulsion and central retinal artery occlusion following rugby injury. Clin Experiment Ophthalmol.

[6] Baker RS, Wilson MR, Flowers CW, Lee DA, Wheeler NC (1996). Demographic factors in a population-based survey of hospitalized, work-related, ocular injury. Am J Ophthalmol.

[7] Reagan DS, Grundberg AB, Reagan JM (2002). Digital artery damage associated with closed crush injuries. J Hand Surg Br.

[8] Scheerlinck TA, Van den Brande P (1994). Post-traumatic intima dissection and thrombosis of the external iliac artery in sportsman. Eur J Vasc Surg.

[9] Sorr EM, Goldberg RE (1975). Traumatic central retinal artery occlusion with sickle cell trait. Am J Ophthalmol.

[10] Vianna RN (1999). Parafoveal arteriolar obstruction after ocular trauma in a patient with systemic lupus erithematosus. Int Ophthalmol.

